# Preventive knowledge, attitude, and vaccination challenges for COVID-19 among Myanmar refugees and irregular migrants in Malaysia

**DOI:** 10.1016/j.jvacx.2023.100360

**Published:** 2023-07-27

**Authors:** Tual Sawn Khai, Muhammad Asaduzzaman

**Affiliations:** a School of Graduate Studies, Lingnan University, Hong Kong Special Administrative Region; b Refugee Law Initiative (RLI), School of Advanced Study, University of London, United Kingdom; c Department of Community Medicine and Global Health, Institute of Health and Society, Faculty of Medicine, University of Oslo, 450 Oslo, Norway

**Keywords:** COVID-19, Malaysia, Myanmar, Irregular migrant workers, Refugee, Vaccination

## Abstract

Access to vaccinations is crucial for everyone in the fight against the COVID-19 pandemic, regardless of migration or refugee status. This study explored COVID-19 prevention knowledge, attitudes, and vaccination challenges among refugees and irregular migrants from Myanmar in Malaysia. This study employed a descriptive mixed-method approach. Convenience sampling was used to conduct an online survey of 174 participants and two focus groups (N = 14). The majority (51.7 %) were refugees, and 48% were irregular migrant workers. In this study, 90.9% of the participants used social networks and social media chats to obtain information about COVID-19, 84.1% understood and found the vaccination campaign helpful, and 44.2% were aware of it. Although 70% of the participants considered vaccination crucial, 95% said that they would not take it because of fear of arrest and deportation, even though they considered vaccination necessary. Approximately 21.2% of the workers reported that their employers did not provide masks or hand sanitisers. Most of the participants (39.7 %) lived in dormitories provided by their employers. This puts them at a higher risk of infection because of the difficulty in practising social distancing. As part of COVID-19 prevention, the government should grant an amnesty period and work with migrant civil society organisations to administer vaccinations and effective measures for all immigrant populations in Malaysia.

## Highlights

• More than 90.9% of respondents relied on Facebook to obtain information about COVID-19, and more than 84.1% understood it.
• Approximately 70% of respondents believe that vaccinations are beneficial if they are free.
• A total of 95% of respondents said they were afraid of arrest if they went out to receive vaccinations.
• 21.2% of respondents do not receive support from their employer for masks and hand sanitisers.
• Approximately 50% of respondents reported difficulty accessing healthcare due to fear of arrest, language barriers and discrimination.

## 1 Introduction

The COVID-19 pandemic has reached unprecedented magnitudes globally, resulting in the loss of over 6 million lives since its outbreak [1]. The most affected groups are migrants and refugees, who face unique challenges due to travel restrictions, xenophobia, poor living conditions, and limited access to healthcare and protection [2]. As of 2020 estimates, 280.6 million of the world population were international migrants, and two-thirds were labour migrants [3]. Again, 82.4 million migrants were forcibly displaced [4], representing another most deprived social group affected by the COVID-19 crisis due to poor sanitation, overcrowded housing conditions, and impractical social isolation [5]. For example, during the earlier COVID-19 outbreak in Singapore, 87.9% of infected cases were reported among migrant workers residing in employer-provided dormitories [6]. Similarly, it has been reported that refugee and migrant populations are at a higher risk because most of them live in crowded shelters or camp-like settings, which often include overcrowding, inadequate sanitation, poor nutrition, and limited health access [7], [8]. In particular, this pandemic has exposed vulnerabilities among irregular migrant workers since they can be arrested at any time by their destination countries' authorities and do not have the right to access healthcare, such as refugees who receive protection under the United Nations [9].

Malaysia has a significant migrant worker population, estimated to be between 2.96 million to 3.26 million, with an estimated 1.23 million to 1.46 million irregular migrants, according to 2017 data [10]. These migrant workers are predominantly low-wage earners and unskilled workers from underdeveloped countries [11] who had to pay a significant toll of socioeconomic loss, morbidity, and mortality during the pandemic [12]. The first COVID-19 case in Malaysia was confirmed on January 25, 2020 [13], [14], following which the government enforced a 14-day Movement Control Order (MCO) to halt the spread of infection under the Prevention and Control of Infectious Diseases Act of 1988 and the Police Act of 1967 [15]. Subsequently, the government conducted raids and detained over 1,368 irregular migrant workers in April 2020 [16]. Furthermore, on 6 May 2020, nearly 29,000 refugees and asylum seekers were arrested for violating the MCO. Some faced penalties of 1,000 Malaysian ringgit (230 USD), while others were detained for nine days [17]. The Malaysian authorities claimed that the operation aimed to prevent irregular migrant workers from travelling to other areas to contain the outbreak [18]; however, they continued to crack down and arrest irregular migrant workers and refugees. Approximately 1,400 irregular migrant workers and refugees, including 98 children and 261 women, were arrested due to invalid visas and other documents as of the end of May 2020 [19]. Subsequently, 49 migrant workers committed suicide, mainly due to fear of detention and their daily hardship to survive in Malaysia between March and October 2020 [20]. Notably, eight of them were Myanmar irregular migrant workers [21].

On the other hand, Mr Khairy, the Minister of Science, Technology and Innovation, stated in February 2021 that “irregular migrant workers will not be arrested when they approach receiving the COVID-19 vaccine when the immunization program begins. Using civil society organizations as partners, we will ensure that they are not detained and that they can come forward freely” [22]. The Malaysian government, however, deported 1,086 Myanmar irregular migrant workers in the same month, despite international organizations and human rights organizations urging the Malaysian authorities not to return these individuals as a military coup had taken place in their home countries [23]. Malaysian authorities have alleged that deported individuals committed immigration offences and were not refugees or asylum seekers. According to the United Nations High Commissioner for Refugees, at least six of those deported have registered, and 17 minors with at least one parent remain in Malaysia [24]. A day before the irregular migrants were deported back to Myanmar, a young woman from Myanmar who had fled to Malaysia due to the civil war committed suicide for fear of being deported back [25]. In May 2021, the Malaysian government announced a two-week nationwide lockdown due to a surge of over 8,000 new COVID-19 cases, with over 61 deaths in a single day [26]. The nationwide lockdown was extended to 28 June because daily COVID-19 cases are still over 5,000 [27]. The Malaysian government continued to deport over 7,200 Indonesian irregular migrant workers in June 2022 [28]. Consequently, irregular migrant workers and refugees were fearful that the government would crack its commitment to vaccinate them without arrest as the crackdown intensified following the enforcement of the nationwide lockdown [22].

Aside from the continued crackdown, this pandemic has brought significant adverse effects, including discrimination and marginalization of refugees and migrant workers. For example, Malaysia Prime Minister Tan Sri Muhyiddin Yasin's statement accused irregular migrant workers caused the “COVID-19 outbreak in Sabah state” [29]. Moreover, even before the COVID-19 pandemic, studies have shown that migrant workers and refugees appear to be at a higher risk of specific disease infection than the host population due to housing conditions, language barriers, and cultural obstacles to accessing health services and are likely to be without any health insurance [30]. In addition, access to healthcare and vaccination was an issue for them due to their inability to pay out of pocket, the discrimination they encountered in public and by healthcare workers, and concerns about their legal status [31], [32], [33], [34]. The inclusion of migrants and refugees in free vaccination programs, regardless of their legal status, is urgently required according to the World Health Organization (WHO) resolution to promote the health of refugees and migrants [35]. The COVID-19 pandemic is a global crisis and everyone cannot be safe until everyone is safe. A recent study also indicated that vaccine demand has increased, and its distribution and marketing have been politicized in response to hegemonic aspirations, causing developing countries to experience difficulty securing vaccines for their citizens, resulting in delays in vaccine delivery to migrants [36]. The Malaysian government announced a free COVID-19 vaccination program for all foreign residents, including students, refugees, and irregular migrants in 2021 [37]. Given Malaysia's ongoing crackdown and arrest, it remains a concern whether irregular migrants and refugees have access to such information and are willing to seek treatment and receive vaccinations. This study aimed to understand the following aspects of Myanmar’s irregular migrant workers (MIMWs) and refugees in Malaysia: (a) knowledge of COVID-19 prevention and associated vaccination information; (b) attitudes toward COVID-19 vaccination and medical treatment if symptoms develop; and (c) practical challenges associated with preventing COVID-19 daily and in the workplace.

### 1.1 Myanmar refugees and migrant workers in Malaysia

Myanmar refugees and migrants work in construction, agriculture, and cleaning in Malaysia, mainly in “3D” jobs (dangerous, dirty, and demeaning) [38]. Malaysia has neither ratified the 1951 Refugee Convention nor ratified the 1967 Protocol. Refugees and asylum seekers are also classified as irregular migrants under Malaysian law [39]. On the other hand, 157,680 refugees from Myanmar registered with the UNHCR, 105,790 Rohingya, 23,290 Chin, and 28,600 other ethnic groups fleeing war and persecution at the end of October 2022 [40]. It was also estimated that at least 17,500 refugees and asylum seekers, including more than 1,500 children, were detained in 21 immigration detention centres across Malaysia without basic amenities [41]. This study defines Myanmar irregular migrant workers as those without legal documents that permit them to work and live in Malaysia. On the other hand, refugees are defined as those who hold valid refugee cards issued by the UNHCR.

## 2 Material and methods

This study was conducted from 25 May to June 20, 2021. This study employed a mixed-method case study approach to understand the knowledge, attitudes, and practical challenges related to the COVID-19 pandemic among Myanmar irregular migrant workers and refugees at their workplaces and in everyday living conditions in Malaysia. The number of Myanmar irregular migrant workers is unknown. Purposive and snowball sampling was employed in this study. Owing to the difficulty of obtaining a visa and the financial limitations of conducting a field study in Malaysia, the author conducted quantitative and qualitative data using online platforms (Zoom), which provide end-to-end encryption. The online survey questionnaire, briefly describing the study objectives, was also sent to different NGOs, refugees, and migrant workers' Facebook pages and groups' chats. The authors relied on non-government organizations (NGOs) referrals to recruit interview participants. Two focus groups (N = 14) were conducted, with some survey respondents who indicated they were willing to participate in the focus groups. Oral consent was obtained from the participants before the focus-group discussion. Myanmar language was used as a means of communication.

### 2.1 Data analysis

Microsoft Excel and IBM SPSS Statistics for Windows, version 25, were used to analyze quantitative data (Chicago, IL, USA). For the qualitative data, the researchers carefully followed the relevant guidelines [42], [43]. The researchers listened to the audio recordings multiple times to familiarize themselves with the participants' stories. The audio recording was transcribed verbatim and translated into English. The transcripts were read and reread line-by-line to understand the text, familiarize themselves with it, and correct any translation errors. The final edited transcript was analyzed using a thematic approach guided by the Knowledge, Attitude, and Practice (KAP) theory to support and compare the quantitative findings.

### 2.2 The framework of the study

**Fig. 1:**
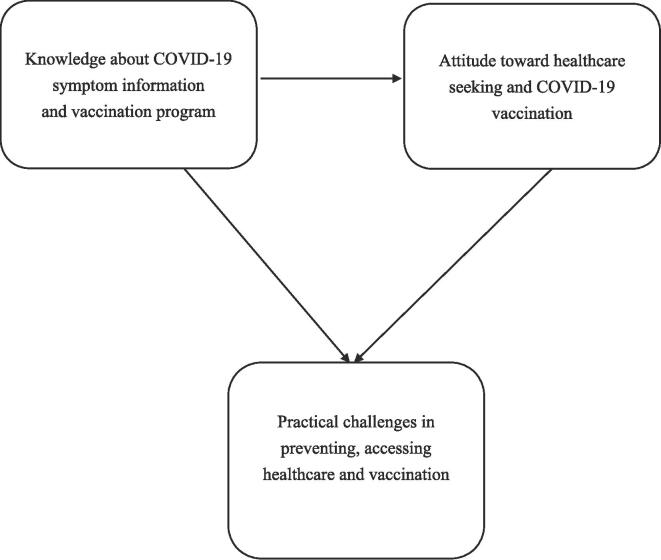
Theoretical Framework Source: Author’s construct.

Knowledge, Attitude, and Practice (KAP) theory guided this research. In the 1960 s, Western scholars proposed the KAP theory as a theory of health behaviour change. Its present human behaviour is categorized into three sequential phases: knowledge acquisition, attitude formation, and behavior formation [44]. Previous studies have used the KAP theory extensively to assess public attitudes toward COVID-19 and identify the most vulnerable population to provide practical intervention recommendations for pandemic mitigation [44], [45]. Following the KAP theory (see Fig. 1), this study examines MIMWs' and refugees' knowledge of COVID-19 prevention information, attitudes toward vaccination and seeking treatment if they develop symptoms, practical challenges to getting COVID-19 vaccination and prevention measures at their workplace, and in their living situations.

## 3 Findings

### 3.1 Social demographics of the participants

A total of 190 individuals provided informed consent and participated in an online survey. Sixteen participants did not complete all the questions. Consequently, only 174 participants were included in the final analysis of this study, of whom 51.7% were refugees and 48% were irregular migrants. There were 79.9% male participants, representing three-quarters of the participants. Nearly half of the participants were between the ages of 25 and 34. Regarding educational attainment, 49.5% had completed high school, 30.5% had completed secondary school, and 9.2 % had completed elementary school.

In the ethnic group, 54.6 % of the participants were Chin ethnic, followed by 21.1 % Bamars and 6.9% Rakhines. Almost half of the participants, 44.8%, lived in Malaysia for over five years. Approximately 39.7% of the participants lived in dormitories with other people, and 37 % lived with family members. Approximately half of the participants worked three days a week and 20.7% were unemployed at the time of the survey. Among the working participants, 39.7% were employed in blue jobs and 14.4% were employed in construction. Table 1 provides a description of the sociodemographic characteristics of the participants.

**Table 1:** Participants' socio-demographic information (N = 174).

**Category**	**Frequency**	**%**
**Gender**		
Male	139	79.9
Female	35	20.1
**Immigration Status**		
Refugee	90	51.7
Irregular migrant worker	84	48.3
**Age**		
18-24	35	20.1
25-34	83	47.7
35-above	56	32.2
**Education level**		
Never been to school	5	2.9
Primary education	16	9.2
Secondary education	53	30.5
High school education	86	49.4
Bachelor degree	11	6.3
Monastic education	2	1.1
Vocational education	1	0.6
**Ethnic group**		
Bamar	42	24.1
Chin	95	54.6
Kacin	3	1.7
Karen	9	5.2
Kayah	1	0.6
Mon	3	1.7
Rakhine	12	6.9
Shan	3	1.7
Rohingya	6	3.4
**Period of working in Malaysia**		
1-2 year	37	21.3
2-3 years	28	16.1
3-4 years	19	10.9
4-5 years	12	6.9
Above 5 years	78	44.8

**Living arrangement**		
Single	39	22.4
Live with other people in dormitory	69	39.7
Live with family	66	37.9

**Employment distribution**		
Construction	25	14.4
Agriculture sector	6	3.4
Hostel and accommodation services	4	2.3
Garment production and sales	5	2.9
Domestic worker	1	0.6
Husbandry related	4	2.3
Seafood processing	3	1.7
Working in restaurant (waiter)	69	39.7
Repair motor vehicles (workshop)	11	6.3
Retail trade and vendor	10	5.7
Currently unemployed	36	20.7

**Current working day per week**		
One day	1	0.6
Two days	22	12.6
Three days	74	42.5
Four days	40	23.5
Five days	36	20.7
Six days	1	0.6

## 4 Knowledge about COVID-19 symptom information and vaccination

Among the survey respondents, 90.9% indicated Facebook as their primary source of information regarding COVID-19 prevention, 33% indicated friends, 11% indicated WhatsApp, 8.5% indicated NGOs, 3.6% indicated the government, and 3% referred to television and newspapers. The interview participants also reported that they typically relied on their respective ethnic communities’ Facebook (group, page, and messenger chat) for information. Furthermore, they mentioned that language barriers to understanding Malaysian, Chinese, and English are significant challenges in obtaining updated information on healthcare precautions.

“I am originally from a small village in Chin State, Myanmar. My educational background was limited to primary education. I cannot read it properly, even in Myanmar language. If I need any information, I must rely on the Facebook page of my ethnic community organization (Zomi Refugee Committee, Zomi Association of Malaysia),” said one participant with refugee status.

Most participants reported understanding that the information they received was valuable for their protection, as 84.1% of participants indicated. In response to the question regarding where to seek medical attention if they developed symptoms, 26.2% cited a hospital, 24.8% cited an NGO health clinic, 18.1% cited a health clinic, 16.8% cited their employer, and only 14.1% referred to a government health clinic authority. Moreover, 44.2% of survey respondents indicated they knew the vaccination program announcement from the government to provide COVID-19 vaccine to irregular migrants and refugees; however, they were unclear about the detailed vaccination schedule and program.

## 5 Attitudes toward healthcare seeking and COVID-19 vaccination

Most of the irregular migrant workers interviewed mentioned that they would seek medical treatment first at their community organization because they fear government authorities due to their irregular status. On the other hand, refugee interview participants also stated that they relied on community healthcare first despite having access to healthcare at a discount in government hospitals, unlike those of irregular migrant worker populations. They are concerned about the cost, and uncertain whether the government will cover their medication costs if they are admitted to the hospital due to COVID-19 symptoms.

**Fig. 2:**
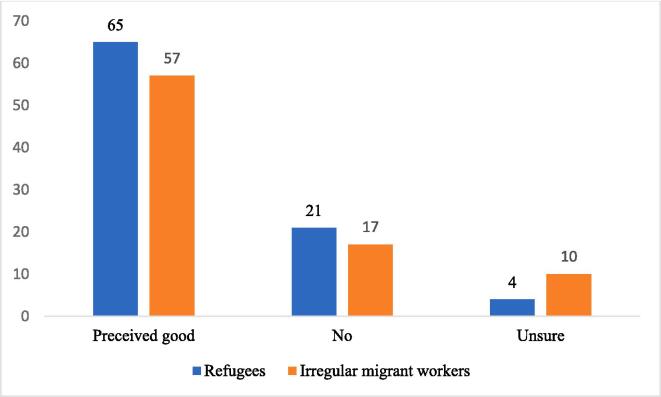
Participant's attitudes toward vaccination.

Regarding vaccination, over 70% of the participants perceived vaccination as necessary if the Malaysian government would grant them freedom and provide them with no arrest threat, whereas 21.8 % did not and 8 % were unsure. Compared to irregular migrant workers, refugees had significantly higher “good perceived” response rates (Fig. 2). Some refugees also stated that they would seek medication if assisted by the United Nations High Commissioner for Refugees (UNHCR). Nevertheless, they had not seen or heard of any individuals receiving assistance from the UNHCR, although they had heard of obtaining support and assistance regarding vaccinations and confirmed cases.

## 6 Practical challenges

### 6.1 Toward COVID-19 precaution measure

#### 6.1.1 Lack of mask and hygiene support

A total of 42.5% of respondents stated they could not afford to purchase personal protective equipment (PPE) to protect themselves from COVID-19 infection. Moreover, 21.2% of those currently employed respondents reported not receiving masks or sanitation from their employers. Furthermore,

“My employer does not provide a mask or hand sanitizer. However, we can occasionally ask if our mask was accidentally damaged or over-sweating because of work. So, it is very challenging to buy masks due to unsafe situations outside and low income,” one participant with refugee status

Specifically, 20.7% of the participants who lost their jobs and were currently unemployed did not receive masks or hand sanitizers from the government or non-governmental organizations.

“It has been a month since I last had a job. To meet my daily needs, I borrow money from others. I did not receive any food, masks, or other additional assistance. I am unable to afford to purchase a mask every day to replace the old mask with a new one. I usually used one mask for one week. Sometimes, I washed and reused it. While I do my part to protect myself if I get infected with COVID-19, I will not seek treatment in the hospital because I do not have legal documents, no money, and cannot speak Chinese, English, or Malaysian languages”, said one of the irregular migrant participants

#### 6.1.2 Work condition

According to the currently employed participants, 54% found it challenging to practice hygiene at work. As required by government guidelines, they wear masks to protect themselves from the spread of COVID-19; however, owing to hot weather and difficulty in breathing, wearing a mask at work is challenging.

“I always wear a mask at work, but it frequently gets sweaty and damp due to the nature of my work. Although my employer provides a mask for workers, it is difficult to request a new mask often within a day.” said one participant with refugee status.

#### 6.1.3 Overcrowded living conditions

**Fig. 3:**
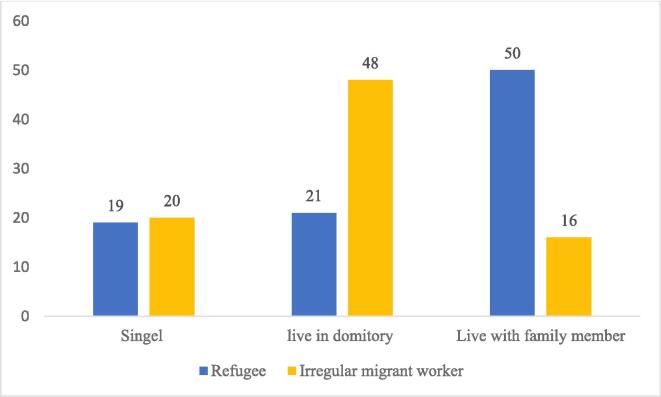
Myanmar irregular migrant workers and refugees' living arrangement status.

In total, 39.7% of the participants are living in other dormitories (Table 1). In particular, irregular migrant workers are likely to live in the dormitory, whereas refugees live with their families (Fig. 3). In most cases, the interview participants expressed concern that if one of us in our dormitory developed COVID-19 symptoms, our lives would be at risk. We could likely all be taken to detention and deported if the authorities heard of it. We do not know how the police will treat us, but we will be detained by a lack of legal status.

“I live together with ten other co-workers in the employer-provided dormitories. I tried my best to prevent COVID-19 infection, but it was very challenging because of the crowded and tiny rooms. If one of us develops symptoms, I have no choice from infection and detention.” said one of the irregular participants.

Some interview participants cited that they had been unemployed for more than two months. They stay with other people in the crowded dormitory to minimize living costs, despite understanding the importance of wearing masks and social distancing for COVID-19 prevention.

### 6.2 Practical challenges toward accessing healthcare

#### 6.2.1 Discrimination in public spaces and transportation problems

Aside from being afraid of being arrested, 16.3% of respondents indicated they had experienced discrimination in public places, while 8.4% had experienced hate speech on public transportation. Furthermore, over 56% of the participants reported difficulty finding a taxi for hiring when going to a health clinic for treatment.

“My daughter was sick. I needed to take her to the clinic. I called a Grab Taxi, but many taxi drivers refused because my destination showed a health clinic. In addition, I hire an interpreter because of language barriers. Since the pandemic, taxis have only been allowed for two people under the law. So, I had to call two taxis, and it was challenging,” Said One participant with refugee status.

#### 6.2.2 Low income, difficulty obtaining sick leave and language barriers

Approximately 70% of survey participants were unemployed or had no regular income. A total of 16.7 % of those currently employed reported having difficulty obtaining sick leave, while 50% reported having difficulty accessing healthcare services. In response to whether they would seek medical treatment if symptoms developed, 42.5% of participants indicated that they would not seek it due to their immigration status, financial hardship, and language barriers. Furthermore, 10.6% of participants reported they were subjected to unpaid labour, whereas 14.4% were threatened with termination if they refused to work overtime.

“If I develop symptoms, I will attempt to cure myself. According to what I read online, many people have recovered despite being infected. To avoid viral infection, warm water with salt is frequently consumed. If I develop symptoms, I will not go to the hospital for treatment unless the cops come and take me there. I feel my life is not safe to go out to seek treatment due to my immigrant status,” said one of the irregular migrant participants.

The majority of interview participants stated that their employers would not cover their medication costs if they developed symptoms related to the cost of treatment. Before the COVID-19 pandemic, some employers covered their workers' medication costs first by deducting later from their salaries payment. However, many employees have been restricted to working three days per week since the COVID-19 outbreak (see Table 1). They are paid daily and do not receive fixed salaries. The income they receive is based only on the number of hours they work, and they find it difficult to manage their everyday living costs.

“Since the pandemic, my employer introduced routine schedules for working days. Most of us work for two days and the remaining two days as a routine because of many business shutdowns and partial opening of the business. I only get around 60 people per day. I hardly earn 1,000 RM (240 USD) per month. I have a hard time surviving. I know how long I will be able to survive in Malaysia. In case I get infected with COVID-19, I will have to endure it on my own because I do not have a legal document, no money, and no social network to help with language interpretation.” said one of the irregular migrant participants.

### 6.3 Practical challenges toward vaccination

#### 6.3.1 Immigrant status and fear of arrest

About receiving COVID-19 vaccination, 95% indicated they feared being arrested and did not feel it safe to travel outside for the vaccination, despite viewing vaccination as a crucial health measure. Similary, the interview participants revealed that authorities conduct operations in many places, especially in the dormitories of irregular migrant populations. Furthermore, we are concerned about the financial costs associated with transportation, and it would be difficult for us to be vaccinated unless the government ceases the crackdown and deportations.

“I am more afraid of being arrested and sent to a detention centre where social distancing is challenging to practice than of contracting COVID-19,” said one participant

Participants suggested that if the Malaysian government has genuine intentions for vaccination, they can arrange for vaccination at their place of employment or community in cooperation with civil society, particularly their ethnic migrant community organizations. Since we do not have legal documents, it is unsafe to go for vaccination, as many people are arrested daily. The government cannot be trusted unless the ongoing crackdown on irregular migrants and refugees ceases. The prospect of being deported back to our home country (Myanmar), particularly under the military regime, terrifies us. Returning home country is unsafe, especially after the military coup.

## 7 Discussions

To the best of the author's knowledge, this is the first study to examine the knowledge, attitudes, and practical challenges related to the COVID-19 pandemic among Myanmar irregular migrants and refugees in Malaysia. The findings of this study clearly show that a large number of MIMWs and refugees received adequate information and knowledge about COVID-19 prevention. More than 70% of the participants perceived vaccination as important; however, 95% feared arrest and indicated not to go for vaccinations in public spaces or hospitals even when they were provided free of charge due to the ongoing raid and detention situation. Specifically, 42.5% of the participants indicated an unwillingness to go to the hospital even if they developed COVID-19 symptoms due to their immigrant status, financial hardship, language barriers, and experience discrimination in public. In studies of migrants and refugees, these barriers have posed significant challenges to vaccination, both before and after the COVID-19 outbreak [46], [47]. Other studies from various countries indicated that immigrants had difficulty accessing healthcare services due to discrimination, language barriers, a lack of interpreters, a lack of legal status, an inability to afford healthcare, unfamiliarity with the doctor, and cultural incompetence [31], [32], [33], [48], [49]. Therefore, allocating a health centre with interpreter services that allow refugees and irregular migrant workers to access healthcare services without discrimination is crucial for the entire refugee and migrant population's vaccination to end COVID-19.

On the other hand, 39.7% of participants in this study lived in dormitories with other people, and most were irregular migrant workers. In line with this study's findings, a previous study reported that 91.1% of migrant workers' accommodations given by their employers in Malaysia do not meet the Worker's Minimum Standards of Housing and Amenities Act 1990 or Act 446 [49]. In particular, migrant workers in the construction sector stay in *Kongsi* houses with approximately 80 occupants [50]. Given that migrant workers live in precarious and overcrowded housing, practising social distancing and maintaining good hygiene is impossible, putting them at a higher risk of contracting COVID-19. However, Malaysian authorities detained over 300 immigrants overnight and imprisoned them to violate the MOC as they gathered, ate, and slept in the same room on June 2021 [51]. Human Rights Watch also reported that Malaysian authorities had escalated the deportations of Myanmar nationals in the wake of the coup, from nearly 2000 to August 2022 [52]. In April 2022, there were at least 17,500 asylum seekers and irregular migrants detained in 21 immigration detention centres across the country, including more than 1,500 children [53]. It has also been reported that Malaysian detention centre facilities are in poor condition despite the COVID-19 epidemic [54]. Meanwhile, a recent study reported a higher likelihood of being brought in dead in the hospital among individuals aged 18 to 59 years, non-Malaysians with no comorbidities, and those not vaccinated against COVID-19 [55]. Therefore, in the context of the global health crisis COVID-19 pandemic, the Malaysian government's massive raids against immigrants were deemed oppressive and inhumane. The continued arrest of immigrants may drive them to hide and impede their efforts to prevent COVID-19. It would be more effective for the Malaysian government to work with civil society organizations and distribute masks, hygiene products, and personal protection equipment to immigrant populations living in dormitories to prevent the spread of COVID-19. Therefore, the Malaysian government must translate the United Nations' statement into action to halt the ongoing crackdown on migrants in the fight against the COVID-19 pandemic [56].

The detention of migrants and refugees in detention centres during the COVID-19 pandemic appears to be a deliberate crime against humanity. A lack of adequate facilities and difficulties in maintaining social distancing and practising adequate hygiene will increase the risk of COVID-19 infection. On the other hand, the study reported that the government in Southeast Asia, including Malaysia, which has become one of the most migrant and refugee destinations, recognized that the country could continue to bear crippling economic restraints associated with movement restrictions because of pandemic prevention measures. The pandemic has drawn attention to the need for increased cooperation and coordination among countries in the region [57], and it is imperative to recognize that international migrant workers and refugees have historically played a crucial role in the economic development of their host countries and countries of origin. For example, the Institute for Democracy and Economic Affairs report in 2019 highlighted that granting refugees the right to work in Malaysia will contribute up to RM 3 billion to the annual GDP and RM 50 million in taxes by 2024 [58]. Thus, the Malaysian government should consider providing amnesty and the right to work, regardless of immigration status, rather than placing them in detention centres.

Furthermore, 60% of participants experienced a reduction in wages, 70% had no regular job, and 10% who were employed also experienced unpaid labour since the outbreak of COVID-19. Of the currently employed participants, 21.2% reported that their employers did not provide them with masks or support for hygiene. In line with this study’s findings, the Malaysian Trades Union Congress (MTUC) stated that “migrant workers were not just unattended in their hostel with no one to turn to, but also faced pay cuts for they did not work during the MOC[59]”. As reported in other Southeast Asian countries [60], participants in this study struggled to meet their living expenses due to job loss and irregular income due to the disruption of the COVID-19 pandemic. They are unable to afford to purchase essential hygiene equipment daily for COVID-19 protection, making them more vulnerable despite adequate preventive measures. Therefore, the Malaysian government and local and international humanitarian organizations urgently need decisive collaborative action to provide basic food, medicine, hygiene, quarantine, and isolation arrangements for those in need.

## 8 Limitations and future research direction

First, the convenience sampling techniques used have limited MIMWs and refugees who do not have access to the survey questionnaire and interviews. Given the limited population size, the study’s findings cannot be generalized to all MIMWs and refugees in Malaysia. Second, the survey questionnaires were distributed online, making it difficult to confirm participants' status reliability. Third, although the author repeated server listening to correct errors, some translation bias may remain in audio transcription from Myanmar to English transcription. Fourth, this study examined MIMWs' and refugees' knowledge of COVID-19 prevention information regarding their consideration of vaccination if they are provided free and healthcare-seeking if they develop symptoms and their daily challenges in practising COVID-19 precautions at work and in their living situations. Therefore, this study did not investigate MIMWs' and refugees' awareness and type of COVID-19 (e.g., fever, malaise, dry cough, and shortness of breath) and knowledge of vaccine side effects. Since this study is limited in scope, future research on the health and well-being of irregular migrants and refugees, mental health, and coping strategies during the COVID-19 pandemic can be considered. In light of these findings, the respective migrant and refugee host governments will be better able to understand how and why the inclusion of these individuals in social protection is more critical than ever, not only in health disasters but also in the event of natural disasters such as earthquakes and climate change.

## 9 Conclusion

Myanmar irregular migrant workers and refugees in Malaysia have a basic understanding of COVID-19 precautions, are positive, and perceive it to be essential to be vaccinated if it is free of charge and there are no arrests. However, the fear of imprisonment and increased public discrimination are significant barriers to vaccination, unless the authorities cease the ongoing raids and detentions. Likewise, job loss, irregular income, salary reductions and nonpayment, poor living conditions, lack of support for food, masks, and hygiene, and inability to afford the necessary personal hygiene equipment make them more vulnerable despite adequate knowledge of COVID-19 prevention measures. Moreover, despite Malaysia returning to normal traditions after two years of lockdown, as in other countries, it is unknown what proportion of irregular migrants, refugees, and asylum seekers have completed their vaccinations. Therefore, the Malaysian government and other stakeholders must provide urgent humanitarian assistance (accessible, acceptable, and available) for these immigrant populations. As this is a global health emergency, no one will be safe until everyone is safe. The United Nations Sustainable Development Goals number 3 explicitly stated: “to ensure healthy lives and promote well-being for all ages.” Therefore, no one should be left behind, neglected, or ignored, regardless of the migrant or refugee status. They must be included in vaccination and humanitarian assistance programs to end the COVID-19 pandemic.

## CRediT authorship contribution statement

**Tual Sawn Khai:** Conceptualization, Methodology, Data curation, Writing – original draft. **Muhammad Asaduzzaman:** Supervision, Validation, Writing – review & editing.

## Declaration of Competing Interest

The authors declare that they have no known competing financial interests or personal relationships that could have appeared to influence the work reported in this paper.

## Data Availability

Data will be made available on request.
